# Effects of Core Stability Training on Deep Stabilizing Muscle Function and Neuromuscular Control

**DOI:** 10.3390/medicina61030364

**Published:** 2025-02-20

**Authors:** Kyeongjin Lee

**Affiliations:** Department of Physical Therapy, College of Health Science, Kyungdong University, Wonju 24764, Gangwon-do, Republic of Korea; kjlee@kduniv.ac.kr

**Keywords:** core stability, muscle activation, rehabilitative ultrasound imaging, pilates

## Abstract

*Background and Objectives*: Pilates-based core stabilization training has garnered increasing attention for its potential to improve deep muscle activation and enhance spinal stability. This study aimed to investigate the effects of Pilates-based core stabilization training on deep stabilizing muscles using rehabilitative ultrasound imaging (RUSI). *Materials and Methods*: A total of 57 healthy adults aged 20 to 29 years were recruited and randomly allocated to either an experimental group (*n* = 29) or a control group (*n* = 28). Participants in the experimental group engaged in Pilates-based core stabilization training three times per week for six weeks, while the control group performed aerobic exercises. The pre- and post-intervention assessments included measurements of muscle thickness, contraction timing, and contraction ratios of the transverse abdominis (TrA), internal oblique (IO), and external oblique (EO) muscles, evaluated using RUSI. *Results*: The experimental group demonstrated significant improvements in TrA and IO thickness (*p* < 0.05), contraction timing (*p* < 0.05), and contraction ratios (*p* < 0.05) compared to the control group. The EO muscle also showed significant, albeit less pronounced, enhancements in thickness and contraction ratios. *Conclusions*: Pilates-based core stabilization training significantly improves core muscle function, including muscle thickness, contraction timing, and contraction ratios. These findings support the inclusion of Pilates exercises in clinical protocols aimed at enhancing core stability.

## 1. Introduction

Musculoskeletal disorders are significant health issues that impose substantial burdens on individuals and societies worldwide. Among these, chronic low back pain is one of the most prevalent conditions experienced by the adult population [1,2]. Chronic low back pain is predominantly classified as nonspecific low back pain, which is closely associated with functional impairments of the primary core muscles responsible for spinal stabilization [3]. Weakness in deep stabilizing muscles such as the transverse abdominis (TrA), internal oblique (IO), and multifidus can compromise spinal stability, leading to altered trunk kinematics and compensatory movement patterns that may exacerbate pain [4]. Studies have shown that delayed or reduced activation of these muscles is associated with abnormal trunk movements during tasks involving postural control and dynamic activities [5]. This weakness is often reflected in daily activities such as lifting objects or transitioning from sitting to standing, where instability may lead to repetitive spinal strain, eventually resulting in chronic low back pain [1]. Consequently, effective core stabilization strategies are essential for maintaining spinal stability and preventing the onset of low back pain [3,6].

Core stability is fundamental for maintaining the stability of the spine and pelvis and enabling efficient bodily movements [7,8]. The TrA plays a critical role by activating prior to movement initiation, thereby stabilizing the spine through a feedforward mechanism [4]. Activation of the TrA reduces the load on the spine, while the IO enhances the rotational stability and lateral support of the trunk. In contrast, the external oblique (EO) primarily contributes to large movements and superficial stabilization [9]. Dysfunction and delayed activation of the TrA and multifidus are frequently observed in individuals with low back pain and are closely linked to deficits in postural control and trunk stability [6,10]. Therefore, the activation and functional recovery of deep stabilizing muscles are pivotal in preventing low back pain and enhancing spinal stability.

Pilates is an exercise method designed to target core stabilization, emphasizing the activation and strengthening of both deep and superficial abdominal muscles [11,12,13]. Specifically, Pilates promotes the independent contraction of TrA and IO while enhancing the coordination of the entire abdominal musculature, including the EO [11]. By incorporating repetitive deep muscle training and the unstable environments provided by apparatuses such as the reformer, Pilates plays a crucial role in improving muscle activation and spinal stability [14,15].

Studies have demonstrated that Pilates-based core stabilization exercises effectively increase the thickness of deep muscles, promote muscle activation, and enhance spinal stability. Research indicates that Pilates-based exercises induce the activation of the TrA and multifidus, increasing the thickness of these muscles [11]. Exercises performed on unstable surfaces, such as the Pilates reformer, further enhance proprioception and muscle activation, contributing to improved balance. Exercises on unstable surfaces using a Pilates reformer have been shown to effectively activate the TrA and IO, leading to improvements in pelvic and trunk stability [15]. Pilates has been found to facilitate the activation of the TrA and play a vital role in coordinating abdominal muscle recruitment patterns [13]. Furthermore, Pilates breathing techniques have been demonstrated to support the functional recovery of deep muscles [12].

To evaluate core muscle activation, previous studies have predominantly utilized fine-wire electromyography (EMG) and surface EMG [16,17]. However, these methods are either invasive or limited to assessing superficial muscles [16]. In contrast, rehabilitative ultrasound imaging (RUSI) offers a non-invasive approach to evaluate the thickness and contraction of deep muscles in real-time, allowing for the precise analysis of muscle state changes pre- and post-exercise [18,19]. Ge L et al. [20] demonstrated that RUSI significantly improved TrA thickness and balance ability in elderly patients with low back pain, emphasizing RUSI’s utility in objectively assessing the effects of core stabilization exercises.

Despite the documented benefits of Pilates-based core stabilization exercises, there remains a lack of systematic and comprehensive analysis examining their effects on key muscle parameters, including the thickness, contraction timing, and contraction ratios of both deep and superficial muscles such as the TrA, IO, and EO. While prior studies have explored individual aspects of muscle activation and thickness using methods like fine-wire EMG and surface EMG [4,16], most of these studies focused on specific exercises or populations without providing an integrated view across multiple variables [3,19]. Moreover, the majority of research has primarily examined clinical populations or acute effects, leaving a gap in understanding the cumulative impact of structured interventions like Pilates-based training on healthy individuals. Therefore, this study addresses the need for a systematic evaluation using non-invasive rehabilitative ultrasound imaging (RUSI) to provide comprehensive insights into core muscle function. Furthermore, research quantitatively evaluating the impact of exercises performed on unstable surfaces on deep muscle activation and spinal stability is limited. This study aims to elucidate the effects of Pilates-based core stabilization training on the deep stabilizing muscles through RUSI, providing a non-invasive and quantitative analysis to clarify the effectiveness of core stabilization exercises.

## 2. Materials and Methods

This study was designed, conducted, and reported in accordance with the Consolidated Standards of Reporting Trials (CONSORT) 2010 statement (Registration number: NCT06672809) [21].

### 2.1. Subjects

This study recruited healthy adults aged 20 to 29 years residing in the Seoul region of South Korea. The recruitment process was conducted through open enrollment at university campuses and local community centers. A total of 94 individuals attended informational sessions, with 60 participants ultimately providing informed consent to join the study. Recruitment strategies involved posting advertisements on campus bulletin boards and organizing informational sessions to clearly communicate the study’s objectives and procedures.

Participants were required to meet the following specific eligibility criteria: to have the physical ability to perform repetitive Pilates movements under guided instruction, a body mass index (BMI) between 18.5 and 25 kg/m^2^, and normal blood pressure levels (systolic blood pressure < 140 mmHg and diastolic blood pressure < 90 mmHg). The exclusion criteria included balance impairments, neurological or musculoskeletal disorders that hinder physical activity, a history of severe cardiovascular events within the last three months, and an inability to commit to the study’s duration. Pregnant individuals and those who had participated in similar research within the previous three months were also excluded.

All participants were provided with comprehensive verbal and written information about the study, including its objectives, procedures, potential risks, and benefits. Researchers explained possible side effects, such as muscle soreness from Pilates exercises, and highlighted the anticipated benefits, such as improved strength and flexibility. Participants were required to sign a written informed consent form before enrollment. They were also informed of their right to withdraw from the study at any time without any consequences. Their personal information was securely managed and protected throughout the study.

The study received ethical approval from the Institutional Review Board of S University (2024-05-013-001) and adhered to the ethical principles outlined in the Declaration of Helsinki. Emphasis was placed on participant safety, rights, and transparency.

### 2.2. Sample Size Calculation

The sample size calculation was conducted using G*Power software (version 3.1.9.7), a robust tool for statistical power analysis across various test designs. The significance level (α) was set at 0.05, reflecting a 5% risk of a Type I error, while statistical power (1-β) was established at 0.80, ensuring an 80% probability of detecting a true effect. The effect size was set as large (η^2^ = 0.146), consistent with Cohen’s guidelines [21].

The analysis indicated that at least 18 participants per group were necessary to achieve sufficient statistical power to detect significant differences. To mitigate the impact of potential attrition during the intervention, an additional 15% of participants were recruited, informed by dropout rates in similar studies. This adjustment led to a final target of 21 participants per group. This approach ensured that statistical power was maintained, allowing for the reliable detection of intervention effects despite potential withdrawals. The sample size calculation also incorporated data from previous studies, ensuring an adequately powered design to explore core muscle activation and functional outcomes effectively.

### 2.3. Procedure

This study adopted a randomized controlled trial design to ensure methodological rigor and systematic implementation. Participants were recruited based on predefined inclusion and exclusion criteria, and informed consent was obtained after providing detailed explanations of the study’s purpose and procedures. Block randomization was used to assign participants to the experimental group or control group, with the randomization process conducted by an independent researcher to minimize selection bias. Initially, 94 participants were recruited for the study. Of these, 34 did not meet the eligibility criteria, leaving a final sample of 60 participants. These participants were randomly allocated to the experimental group (*n* = 30) and control group (*n* = 30) using a computer-generated randomization process.

Pre-intervention assessments were conducted within one week prior to the start of the intervention. These assessments included demographic data, baseline characteristics, and muscle function evaluations. Muscle function was assessed using RUSI to precisely measure the thickness and activation timing of abdominal muscles, including the TrA, IO, and EO. This baseline assessment ensured homogeneity between groups and provided a reference point for subsequent analyses.

The intervention phase lasted for six weeks. Participants in the experimental group underwent Pilates-based core stability training. These sessions were conducted three times per week, with each session lasting 50 min. The training utilized a Pilates reformer and focused on exercises performed on unstable surfaces to enhance deep muscle activation, particularly targeting the TrA. A variety of exercises designed to activate deep core muscles were included throughout the intervention period. In contrast, participants in the control group engaged in aerobic exercises, such as walking. These sessions were also conducted three times per week, with each session lasting 50 min. The intensity of the aerobic exercises was progressively increased over the six-week period. Both groups followed identical schedules to ensure consistency in training duration and frequency.

**Figure 1:**
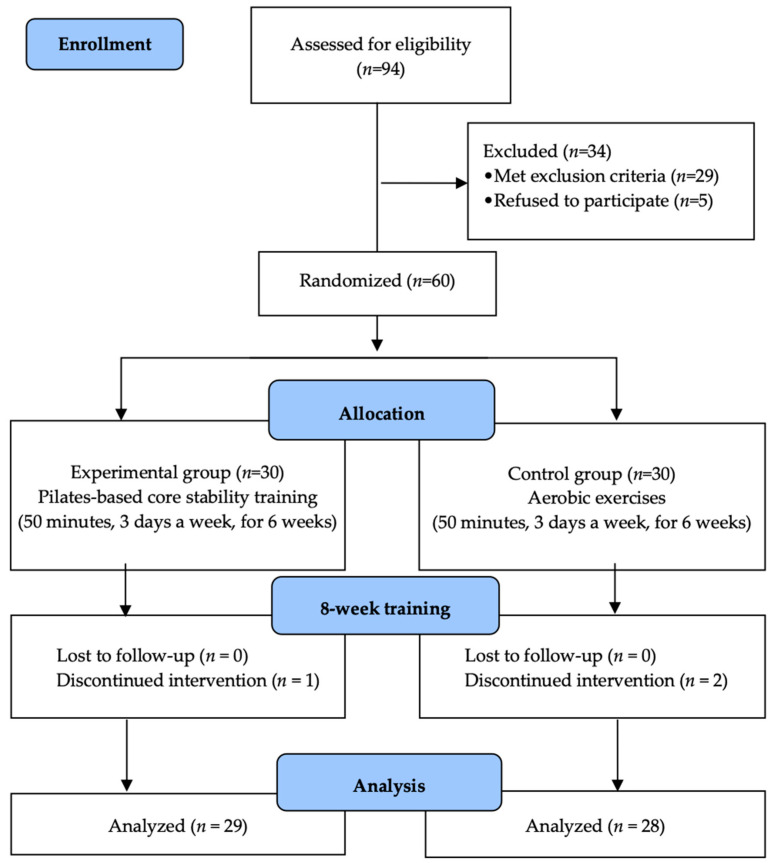
Flow diagram of the study.

Post-intervention assessments were conducted within one week following the completion of the intervention phase. These assessments were performed using the same methods as the pre-intervention evaluations. Data collection and analysis adhered to standardized protocols to ensure reliability and reproducibility. Participants who withdrew due to health-related reasons or attended fewer than 80% of the intervention sessions were excluded from the final analysis. Data from the excluded participants were documented and analyzed through an attrition analysis, detailing the reasons for and patterns of withdrawal. Ultimately, 29 participants in the control group and 28 in the experimental group completed the study, and their pre- and post-intervention data were analyzed. To enhance the study’s reliability, the evaluators conducting the pre- and post-intervention assessments were blinded to group allocations (Figure 1).

### 2.4. Intervention

Participants in the experimental group performed a Pilates-based core stabilization training program designed to enhance deep muscle activation and spinal stability, while participants in the control group engaged in aerobic exercise. Both interventions were conducted three times per week for six weeks, with each session lasting 50 min. Each session consisted of warm-up, main exercise, and cool-down phases.

The experimental group utilized the Pilates reformer (V2 MaxTM Reformer, Toronto, ON, Canada) for core exercises. Each session was structured as 10 min of warm-up, 35 min of main exercise, and 5 min of cool-down. Exercises were performed on an unstable surface to target the activation of deep stabilizing muscles such as the TrA. The program was designed to progressively increase in intensity and complexity over the six weeks. During the initial three weeks, participants performed exercises with low spring resistance, focusing on the basic activation and stabilization of deep muscles. From the fourth week onward, spring resistance and movement complexity were increased to enhance deep muscle integration and whole-body coordination.

The warm-up phase included breathing exercises combining diaphragmatic breathing with arm movements to prepare the trunk for stabilization. Participants performed pelvic tilts in a supine position to gently activate the TrA and IO. Spinal stretches, such as sitting on the reformer and leaning forward to stretch the back and legs, were also included (Table 1).

During the first three weeks of the main exercise phase, the focus was on enhancing deep muscle activation and stabilization. Participants performed movements such as footbar pushes and pulls to activate the legs and TrA, as well as performing the hundred exercise at a low spring resistance to strengthen the TrA. Bridge exercises involved lifting and lowering the pelvis to strengthen the gluteal and abdominal muscles, while leg circles were included to improve hip stability. Side-lying exercises using the footbar targeted the leg muscles, and kneeling carriage pushes activated the TrA and IO. Single thigh stretches on the reformer were also included to enhance pelvic stability and TrA activation.

From the fourth to sixth week, the main exercise phase emphasized deep muscle integration and whole-body coordination. Participants performed advanced exercises such as the snake and twist to strengthen deep abdominal muscles and spinal stability through rotational movements. Shoulder bridge exercises incorporating leg extensions targeted the TrA and gluteal muscles. Prone swimming preps, alternating arm and opposite leg lifts, were included to enhance TrA activation and coordination. The leg spring series involved attaching the springs to the legs and performing lifts to strengthen the TrA and IO. Other exercises included swan dive movements in a prone position to activate the TrA and spinal extensors, and the short box round back exercise, which involved rounding the upper body on the reformer to target the TrA and IO. Long stretch exercises, involving plank positions with reformer movements, were also included to activate the TrA and IO.

The cool-down phase featured child’s pose to relax the lower body and abdominal muscles, as well as cat-cow stretches in a quadruped position to enhance spinal stability and alleviate tension.

The control group performed aerobic exercise in the form of walking. Each session included 10 min of warm-up, 35 min of moderate-intensity aerobic exercise, and 5 min of cool-down. Exercise intensity was progressively increased over the three weeks, ensuring that the participants performed within a pain-free range.

Attendance was recorded for all participants, with a minimum attendance rate required for inclusion in the analysis. Sessions were supervised by trained physical therapists to ensure proper exercise execution and adherence to the protocol. All participants were instructed to report any adverse effects or difficulties during the intervention, and sessions were discontinued immediately if any issues arose.

### 2.5. Outcome Measure

The thickness of the abdominal muscles was assessed using RUSI (Xcube60, Alpinion, Alpinion Medical Systems Co., Ltd., Seoul, Republic of Korea), a widely recognized, non-invasive method noted for its high reliability [22]. To ensure precision and consistency in measurements, the ultrasound operators in this study received training based on standardized protocols established in prior validated studies. The reliability of RUSI for assessing TrA, IO, and EO has been extensively demonstrated through inter-rater and intra-rater reliability assessments reported in the literature [22]. Measurements were conducted for the TrA, IO, and EO muscles in both resting and contracted states to determine muscle activation levels. Each muscle’s thickness was measured three times per participant, and the median value was used for analysis to ensure precision. The specific measurement sites were as follows: the TrA was measured at the midaxillary line, equidistant between the iliac crest and the costal margin; and the IO and EO were measured at the same location, utilizing fascial boundaries as anatomical landmarks. For the rectus abdominis, measurements were taken 3 cm above the umbilicus and 3 cm lateral to the midline. Comparisons of muscle thickness during resting states and abdominal drawing-in maneuvers (ADIM) facilitated the calculation of contraction ratios, derived using the following formula:

[(thickness during contraction − thickness at rest)/thickness at rest] × 100

Muscle contraction timing was evaluated to determine the response speed of muscles to rapid movements, utilizing synchronized surface EMG and ultrasound M-mode imaging. This integrated approach enabled the simultaneous recording of the initiation of muscle activation and dynamic changes, ensuring the precise assessment of muscle response timing. The analysis focused on the feed-forward activation (preparatory activation) of the TrA, IO, and EO muscles. During tasks involving upper limb movements (e.g., shoulder flexion) or ADIM, the onset of activation for each muscle and the duration required to reach peak contraction were recorded. Each measurement was repeated 3–5 times, with the median value used to ensure reliability and accuracy in the findings.

### 2.6. Statistical Analysis

The normality of the data was assessed using the Shapiro–Wilks test, and the results indicated that all demographic data and other study variables followed a normal distribution. Descriptive statistics were utilized to summarize all data, with variables reported as the mean and standard deviation. To compare dependent variables between groups, independent t-tests were employed to evaluate the mean differences for continuous variables, while chi-square tests were chosen to analyze the distribution differences for categorical variables. Additionally, paired *t*-tests were used to compare changes in the dependent variables within groups. Statistical significance was set at a *p*-value of ≤0.05. All statistical analyses were performed using SPSS version 25.0 (IBM Corporation, Armonk, NY, USA).

## 3. Results

The general characteristics of the subjects in the experimental and control groups, as shown in Table 2, were analyzed using the appropriate statistical tests to ensure homogeneity. No statistically significant differences were observed between the groups in terms of age, height, weight, BMI, or gender distribution. These findings confirm that the two groups were homogeneous and comparable.

### 3.1. Muscle Thickness

This study investigated the changes in the muscle thickness of the TrA, IO, and EO in resting and contracted states between the experimental and control groups (Table 3). The findings demonstrated significant differences favoring the experimental group across all measured variables.

For the TrA at rest, the experimental group showed a significant increase in muscle thickness from pre- to post-intervention (t = 14.208, *p* < 0.001), while the control group did not exhibit significant changes (t = 1.382, *p* = 0.179). The pre–post changes between the groups were significantly different (*p* < 0.001). During contraction, the experimental group demonstrated a significant increase in TrA thickness (t = 16.159, *p* < 0.001), whereas the control group did not show any significant changes (t = 0.236, *p* = 0.815). The differences in the pre–post changes between the two groups were statistically significant (*p* < 0.001).

For the IO at rest, the experimental group exhibited a significant increase in muscle thickness (t = 16.042, *p* < 0.001), while the control group showed no significant change (t = 1.461, *p* = 0.452). The between-group comparison of the pre–post changes revealed a significant difference (*p* < 0.001). During contraction, the experimental group also displayed a significant increase in IO thickness (t = 8.223, *p* < 0.001), while the control group showed no significant difference (t = 1.061, *p* = 0.316). The pre–post differences between the groups were statistically significant (*p* < 0.001).

For the EO at rest, the experimental group showed a significant increase in thickness (t = 6.634, *p* < 0.001), whereas the control group did not demonstrate significant changes (t = 0.900, *p* = 0.379). The between-group differences in the pre–post changes were significant (*p* < 0.001). During contraction, the experimental group exhibited a significant increase in EO muscle thickness (t = 11.446, *p* < 0.001), with no significant changes observed in the control group (t = 0.798, *p* = 0.432). The differences in the pre–post changes between the groups were statistically significant (*p* < 0.001).

### 3.2. Muscle Contraction Time and Muscle Contraction Ratios

This study analyzed the changes in contraction time and muscle contraction ratios of TrA, IO, and EO between the experimental and control groups (Table 4). The findings revealed significant improvements in the experimental group across most parameters compared to the control group.

For contraction time, the experimental group showed a significant decrease from pre- to post-intervention (t = 9.869, *p* < 0.001), while the control group did not exhibit a significant change (t = 1.888, *p* = 0.070). The pre–post differences between the two groups were statistically significant (*p* < 0.001).

For the TrA contraction ratio, the experimental group demonstrated a significant increase from pre- to post-intervention (t = 5.944, *p* < 0.001), whereas the control group did not show any significant change (t = 0.335, *p* = 0.738). The between-group comparison of the pre–post changes was statistically significant (*p* < 0.001).

For the IO contraction ratio, the experimental group exhibited a significant increase from pre- to post-intervention (t = 2.986, *p* = 0.006), while the control group showed no significant change (t = 0.853, *p* = 0.401). The differences in the pre–post changes between the two groups were significant (*p* = 0.002).

For the EO contraction ratio, neither the experimental group (t = 3.565, *p* = 0.002) nor the control group (t = 0.577, *p* = 0.571) showed significant changes in the pre–post differences. Additionally, the between-group comparison was not statistically significant (*p* = 0.043).

## 4. Discussion

Core muscles play a pivotal role in spinal stability and the prevention of low back pain, with the activation of the TrA and IO being critical for enhancing neuromuscular control and improving postural regulation. The findings of this study demonstrated significant short-term improvements in the thickness, contraction timing, and contraction ratio of TrA, IO, and EO in the experimental group, supporting the potential efficacy of Pilates-based exercises.

This study revealed significant increases in the thickness of TrA, IO, and EO following Pilates-based core stabilization training. Specifically, TrA showed an increase of 0.06 cm at rest and 0.14 cm during contraction, while the IO thickness increased by 0.11 cm at rest and 0.20 cm during contraction. These results underscore the effectiveness of Pilates-based exercises and align with prior research. Shamsi M et al. [3] reported a 126% increase in TrA thickness after core stabilization exercises, highlighting the correlation between increased muscle thickness, spinal stability, and reduced LBP symptoms. Similarly, Ge L et al. [20] found a 40–70% increase in TrA thickness during ADIM, which is consistent with this study’s observed improvements. These findings suggest that Pilates exercises may employ mechanisms similar to ADIM to effectively activate and strengthen the TrA. Ehsani F et al. [19] reported a 143% increase in TrA thickness among healthy individuals, compared to a 109% increase in LBP patients, emphasizing the impaired TrA activation in individuals with LBP. The results of this study further validate the potential of Pilates exercises to address these impairments. Moreover, the observed increases in IO thickness align with the findings from Tsartsapakis I et al. [23], who identified Bird Dog as being particularly effective in enhancing IO thickness. The alignment between the IO thickness increase (0.20 cm) observed in this study and Ioannis’ findings demonstrates the potential of Pilates exercises to activate deep muscles in unstable conditions. For EO, although this study recorded significant thickness increases, the changes were relatively smaller than those for TrA and IO. This observation can be attributed to EO’s primary function as a superficial muscle involved in larger movements and superficial stabilization. These findings align with Gibbons TJ Bird ML [9], who reported EO’s structural role in force transmission during gross motor tasks, highlighting its limited adaptation to exercises targeting deep muscle activation.

Pilates-based exercises significantly reduced the contraction timing of TrA and IO, indicating improved neuromuscular control of these muscles. These findings are particularly relevant for addressing dysfunctions commonly observed in individuals with LBP. Vasseljen O et al. [1] reported significant delays in TrA contraction timing during shoulder flexion in individuals with LBP, attributing this delay to diminished feedforward activation. This study’s findings demonstrate that Pilates-based exercises can effectively reduce such delays. Similarly, Ge L et al. [20] identified a 15–20 ms delay in TrA activation among LBP patients, which this study confirmed could be mitigated through progressive core stabilization training. By employing a Pilates reformer, this study introduced a dynamic and unstable environment that likely enhanced neuromuscular adaptation and improved TrA contraction timing. Ehsani F et al. [19] observed significant delays in TrA and EO contraction timing in LBP patients, linking these delays to the impaired motor control of deep abdominal muscles. The 3.55 s reduction in TrA contraction timing observed in this study supports the efficacy of structured Pilates programs in restoring neuromuscular control. Furthermore, the reduction in IO contraction timing aligns with Sitilertpisan P et al. [24], who reported improved IO activation during dynamic tasks, suggesting enhanced coordination among the abdominal muscles. Although EO exhibited less pronounced improvements in contraction timing, this is consistent with its role as a superficial stabilizer, being less sensitive to exercises targeting deep core activation. Gibbons TJ Bird ML [9] highlighted EO’s primary role in force generation, which may explain its limited responsiveness to Pilates exercises emphasizing deep stabilization.

This study also demonstrated significant improvements in the contraction ratios of TrA and IO, reinforcing the role of Pilates exercises in enhancing muscle activation and spinal stability. Shamsi M et al. [3] reported a 1.61-fold increase in TrA contraction ratios following core stabilization exercises, linking these improvements to reduced LBP symptoms and enhanced spinal stability. Similarly, this study observed notable increases in TrA contraction ratios, confirming the effectiveness of Pilates exercises in strengthening deep stabilizing muscles. Sitilertpisan P et al. [24] highlighted the superior efficacy of dynamic stabilization exercises like Bird Dog and Side Plank in improving TrA and IO contraction ratios. While this study shared similar findings, it also emphasized the unique contributions of Pilates reformer exercises performed on unstable surfaces, providing a systematic approach to enhancing muscle activation and spinal stability. These findings underscore the multifaceted benefits of Pilates exercises in improving dynamic core stability. EO also showed significant improvements in contraction ratios, though these were less pronounced compared to TrA and IO. Gibbons TJ Bird ML [9] noted that EO predominantly contributes to gross motor tasks, explaining its limited responsiveness to exercises focusing on deep core activation. Despite this, the study’s findings highlight EO’s important role in supporting overall core functionality.

This study provides compelling evidence that Pilates-based core stabilization training significantly improves the thickness, contraction timing, and contraction ratios of TrA, IO, and EO. By utilizing RUSI, this research offers an objective and quantitative assessment of muscle activation and spinal stability. The findings extend the existing literature, demonstrating the potential of Pilates exercises as a preventive and therapeutic intervention for LBP. However, the feasibility of implementing Pilates in clinical settings involves certain limitations that should be considered. Challenges such as the need for trained instructors and ensuring patient adherence may influence the effectiveness of the intervention. Future studies should focus on developing strategies to address these challenges and assess the practical application of Pilates-based training in rehabilitation settings. Additionally, further research is necessary to validate these findings by exploring the long-term effects of Pilates exercises across diverse populations and exercise environments.

## 5. Conclusions

This study underscores the effectiveness of Pilates-based core stabilization training in enhancing the function of key core muscles, including the TrA, IO, and EO, among healthy young adults. By improving muscle thickness, contraction timing, and contraction ratios, this training method, conducted on unstable surfaces such as the reformer, proved effective in promoting deep muscle activation and enhancing spinal stability. RUSI served as a precise and non-invasive tool to evaluate these improvements, further validating the clinical relevance of Pilates exercises. These findings position Pilates-based training as a robust preventive and therapeutic approach for core stability.

## Figures and Tables

**Table 1:** Pilates-based core stabilization training.

Phase	Exercises	Exercise Protocol
Warm-up	Breathing with Arm Movement	In a supine position, perform diaphragmatic breathing while moving the arms up and down to prepare for abdominal and trunk stabilization.
	Pelvic Tilts	In a supine position, move the pelvis forward and backward to gently activate abdominal muscles.
	Spine Forward Stretch	Sit on the reformer with legs extended forward and bend the upper body to stretch the back and legs.
Main Exercise (1–3 weeks)	Footwork	Push the footbar by extending the legs, maintaining pelvic and spinal stability, and return by bending the knees.
	Hundred	Pump the arms in a supine position with the legs in tabletop, coordinating with 5 breaths in and 5 breaths out for 10 sets, to enhance core stability and endurance.
	Bridge	Lift and lower the pelvis sequentially from the tailbone to the upper spine while maintaining pelvic and spinal stability.
	Leg Circles	Perform controlled circular leg movements while maintaining pelvic stability in an imprinted position, focusing on hip joint mobility and core control.
	Sleeper	In a side-lying position, maintain pelvic stability while pressing the top leg against the footbar in alignment.
	Knee Stretch	In a kneeling position, maintain a neutral spine while pushing and pulling the carriage back and forth with the legs, focusing on stabilizing the pelvis and trunk.
	Single Thigh Stretch	In a standing position with one foot on the floor and the other on the shoulder rest, maintain a neutral spine while pushing the carriage by extending the front leg, ensuring pelvis and trunk stability throughout.
Main Exercise (4–6 weeks)	Snake and Twist	Place one hand on the footbar and the other on the carriage, with the feet supported on the edge of the footbar. In a side-facing position, maintain a neutral spine while extending the legs and pushing the carriage. Focus on maintaining alignment during spinal articulation, and use the trunk and pelvis to return to the starting position.
	Frog	With the feet in straps, extend and bend the legs in a diamond shape while maintaining pelvic stability, targeting lower limb strength and enhancing hip flexibility.
	Shoulder Bridge	Lie in a supine position with the knees bent. Lift the pelvis while maintaining a neutral spine, stabilizing the trunk and pelvis. Alternate leg extensions during the movement.
	Side Split	Stand with one foot on the platform and the other on the carriage. Maintain pelvic and trunk stability while pushing the carriage outward through hip abduction, ensuring equal weight distribution on both legs, and return to the starting position.
	Swimming (Long Box)	Lie prone on the box with the arms and legs extended. Alternately lift the opposite arm and leg while maintaining trunk stability.
	Swan Dive (Long Box)	In a prone position on the box, extend the spine by lifting the chest and legs while maintaining alignment and stabilizing the shoulders and pelvis.
	Round Back (Short Box)	Sit on the box with the feet secured in foot straps. Curve the spine into a C-shape while maintaining pelvic stability and flexing the trunk forward and backward to enhance spinal mobility and core engagement.
	Long Stretch	Assume a plank position with the feet on the headrest and hands on the footbar. Maintain a straight alignment of the body while pushing the carriage forward and pulling it back to strengthen the core and stabilize the pelvis and trunk.
Cool-down	Child’s Pose	Relax the lower back and abdomen.
	Cat and Cow Stretch	Perform the flexion and extension of the spine in a quadruped position to enhance stability and reduce tension.

**Table 2:** General characteristics of subjects.

	Experimental Group (*n* = 29)	Control Group (*n* = 28)	*χ*^2^/*t*	*p*
Age (year)	24.00 ± 2.19	23.75 ± 2.46	0.406	0.686
Height (cm)	169.62 ± 7.24	168.82 ± 7.28	0.416	0.679
Weight (kg)	65.10 ± 6.98	63.93 ± 7.51	0.612	0.543
BMI (point)	22.56 ± 0.98	22.34 ± 0.93	0.876	0.385
Gender (male/female)	16/13	14/14	0.696	0.153

BMI = body mass index. Values are expressed as mean ± standard deviation.

**Table 3:** The changes in muscle thickness.

		Experimental Group (*n* = 29)	Control Group (*n* = 28)	*t*	*p*	CI for Difference		Effect Size	MDC (%)
						Lower	Upper		
TrA (rest)	Pre	0.41 ± 0.02	0.41 ± 0.02	0.285	0.777				
	Post	0.46 ± 0.03	0.41 ± 0.03						
	Pre–Post	0.06 ± 0.02	0.01 ± 0.03	6.622	0.000	0.03	0.06	1.75	0.01
	*t*	14.208	1.382						19.51
	*p*	0.000	0.179						
TrA (contract)	Pre	0.59 ± 0.02	0.60 ± 0.03	1.183	0.242				
	Post	0.73 ± 0.06	0.60 ± 0.06						
	Pre–Post	0.14 ± 0.05	0.00 ± 0.06	9.378	0.000	0.11	0.17	2.48	0.02
	*t*	16.159	0.236						17.15
	*p*	0.000	0.815						
IO (rest)	Pre	0.92 ± 0.04	0.88 ± 0.12	1.574	0.121				
	Post	1.03 ± 0.05	0.88 ± 0.13						
	Pre–Post	0.11 ± 0.04	−0.01 ± 0.04	10.964	0.000	0.10	0.14	2.90	0.00
	*t*	16.042	0.764						0.00
	*p*	0.000	0.452						
IO (contract)	Pre	1.06 ± 0.03	1.03 ± 0.11	1.528	0.132				
	Post	1.26 ± 0.14	1.01 ± 0.15						
	Pre–Post	0.20 ± 0.13	−0.02 ± 0.07	7.861	0.000	0.17	0.28	2.08	0.07
	*t*	8.223	1.461						33.71
	*p*	0.000	0.156						
EO (rest)	Pre	0.75 ± 0.05	0.76 ± 0.05	0.625	0.535				
	Post	0.82 ± 0.06	0.75 ± 0.06						
	Pre–Post	0.07 ± 0.06	−0.01 ± 0.04	5.968	0.000	0.05	0.10	1.58	0.00
	*t*	6.634	0.900						0.00
	*p*	0.000	0.377						
EO (contract)	Pre	0.79 ± 0.03	0.81 ± 0.04	1.980	0.053				
	Post	0.90 ± 0.06	0.80 ± 0.06						
	Pre–Post	0.11 ± 0.06	−0.01 ± 0.04	9.624	0.000	0.09	0.14	2.55	0.00
	*t*	11.046	0.798						0.00
	*p*	0.000	0.432						

TrA = transverse abdominal muscle; IO = internal oblique muscle; EO = external oblique muscle; CI = confidence interval; MDC = minimal detectable change. Values are expressed as mean ± standard deviation.

**Table 4:** The changes in the contraction timing and muscle contraction ratio.

		Experimental Group (*n* = 29)	Control Group (*n* = 28)	*t*	*p*	CI for Difference		Effect Size	MDC (%)
						Lower	Upper		
Time (s)	Pre	18.10 ± 1.45	18.07 ± 1.33	0.087	0.931				
	Post	14.55 ± 1.99	18.15 ± 1.51						
	Pre–Post	−3.55 ± 1.94	0.08 ± 0.80	9.188	0.000	−4.43	−2.84	−2.43	1.00
	*t*	9.869	0.542						28.09
	*p*	0.000	0.592						
TrA (Ratio)	Pre	44.28 ± 3.49	47.15 ± 9.32	1.551	0.127				
	Post	57.23 ± 10.33	45.25 ± 16.69						
	Pre–Post	12.95 ± 11.73	−1.90 ± 20.79	3.335	0.002	5.92	23.77	0.88	0.00
	*t*	5.944	0.483						0.00
	*p*	0.000	0.633						
IO (Ratio)	Pre	15.45 ± 3.69	16.71 ± 4.11	1.219	0.228				
	Post	22.34 ± 11.98	15.13 ± 9.97						
	Pre–Post	6.88 ± 12.41	−1.58 ± 10.11	2.817	0.007	2.44	14.48	0.75	6.39
	*t*	2.986	0.828						92.82
	*p*	0.006	0.415						
EO (Ratio)	Pre	5.85 ± 6.02	6.98 ± 2.86	0.900	0.372				
	Post	10.55 ± 6.40	7.55 ± 8.62						
	Pre–Post	4.70 ± 7.09	0.57 ± 7.99	2.067	0.043	0.13	8.14	0.55	3.65
	*t*	3.567	0.375						77.70
	*p*	0.001	0.711						

TrA = transverse abdominal muscle; IO = internal oblique muscle; EO = external oblique muscle; CI = confidence interval; MDC = minimal detectable change. Values are expressed as mean ± standard deviation.

## Data Availability

The data presented in this study are available on request from the corresponding author.
